# Explaining the Factors Influencing the Individuals’ Continuance Intention to Seek Information on Weibo during Rainstorm Disasters

**DOI:** 10.3390/ijerph17176072

**Published:** 2020-08-20

**Authors:** Sheng Cheng, Liqun Liu, Ke Li

**Affiliations:** 1 School of Journalism and Communication, Wuhan University, Wuhan 430072, China; chengsheng@whu.edu.cn (S.C.); worbych@126.com (K.L.); 2 Center for Studies of Media Development, Key Research Institute of Humanities and Social Sciences at Universities, Ministry of Education, Wuhan University, Wuhan 430072, China

**Keywords:** risk communication, risk management, Weibo, rainstorms, satisfaction, continuance intention, expectation–confirmation model

## Abstract

Being an interactive process, the success of risk communication needs to ensure the individuals’ right to know and influence their attitudes and perceptions of risk. Ubiquitous social media have expanded risk communication channels and innovated ways of risk communication. At the same time, uncertainty also arises with the diversity and variety of social media. Taking the rainstorm disaster in China as an example, this study focuses on factors affecting the individuals’ continuance intention of information seeking on Weibo (a social media platform similar to Twitter). Based on 377 valid respondents, this study applied an extended expectation–confirmation model (ECM), from which the results of partial least squares structural equation modeling (PLS-SEM) suggested that continuance intention is positively influenced by factors including effort expectancy, social influence, facilitating conditions, and satisfaction. Among them, satisfaction contributes the most, which helps maintain a balance between performance expectancy and continuance intention. Taking the individuals’ continuance intention to seek information on Weibo as the clue, this research provides government agencies with practical advice on how to use social media for more efficient risk communication during disasters and establish emergency preplans to respond to natural disasters.

## 1. Introduction

Natural disasters are threatening people’s life and property. They are practically beyond human control. Uncertainty is the most prominent feature of natural disaster risk. It has negative effects on the sustainable development of both the environment and mankind with its disastrous, and its consequences including human life loss, economic loss, and the adverse impact on the ecological environment.

The essence of risk communication has shifted from one-way messages in the past to empower the nonexperts, that is, placing the nonexperts in the position of actively participating in the communication process. Due to the status transformation of the nonexperts, “interactivity” has become the key factor in determining whether the information can be effectively accepted in choosing the risk communication path. Risk communication acts as an exchange system in which individuals, groups, and even institutions share information and opinions. As a result, this system does not only include multiple messages related to the nature of risk but also other relevant information.

The success of risk communication lies in raising the public’s understanding of relevant issues or actions and the satisfaction obtained by those involved after being adequately informed within the limits of available knowledge [1]. Effective risk communication can reduce the risk of natural disaster from two aspects. On the one hand, effective risk communication can prevent the spread of adverse effects of natural disasters [2]. On the other hand, it can reduce the damage of natural disasters to the economy and society directly by improving the efficiency of disaster relief.

The emergence and widespread use of social media bring new uncertainty to risk communication. At present, social media has become a communication platform for the government, the public, media, enterprises, organizations, and other entities. During emergencies including natural disasters, all kinds of social media serve as active information platforms for victims to seek and share disaster information [3]. The innovative and effective application of social media during emergencies increased the public’s awareness and initiative in the process of disaster recovery. On the other hand, it has also altered the way officials manage disasters. In consequence, situational awareness, emotional relief, and acknowledgment of efforts are all improved [4].

There is an increase in studying the application of social media in natural disasters. Ngamassi, Ramakrishnan, and Rahman [5] identified some major reasons for the public to use social media during disasters: Seeking information, receiving timely information, obtaining unique information, determining disaster magnitude, checking in with family and friends, self-mobilizing, and seeking emotional support and healing.

Researches in this field usually encompass two dimensions: The functional level, and the multi-faceted content.

Researches on the functional level summarize the role played by social media in preventing and mitigating disasters. Communication activities during the disaster mainly include information collection and dissemination, relief goods collection and flow, organization of manpower and task dispatch, expression of mood and expression of support. Alexander [6] concluded that the emergency field used social media for the following seven purposes: Getting access to public debate, being able to monitor situations, effectively and immediately responding to and managing emergencies, maintaining crowd-sourcing, promoting collaboration and social cohesion, holding charitable donation, and boosting research. As the government plays a leading role in risk governance, social media is a low-cost policy tool and channel. Meanwhile, social media helps the public form spontaneous information communication channels, ways of communication and emergency functions. Through the cooperation between individuals, the collective intelligence superior to any individual will be generated.

The second dimension concerns the multi-faceted content gathered on social media, which can innovate the strategies of traditional risk communication. The most basic is the collection of social media data. PetaJakarta.org project (now has been replaced by PetaBencana), a highly-praised project, pioneered real-world implementations by applying crowdsourced geospatial data to the area of modern disaster management [7]. On the level of data collection, efficiency improvement of risk communication is taken as an important indicator. After exploring the frequency of generic and specific terms related to food-security, and quantifying the way in which network size is related to Twitter activity in disasters, Niles et al. [8] suggested an ideal social contagion in which common people are engaged and plays a vital role in risk communication. To check the role of extracted information in informing disaster responses and recovery, Fang et al. [9] put forward a framework for the evaluation of disaster impacts based on data collected from Weibo. In addition, by combining with geographic information data, social media data can better assist disaster prevention and mitigation work [10]. For journalists, they can also use the event detection method to integrate the data available on social media into their news stories [11].

However, using social media during emergencies faces challenges, including: The identification of important situational information; the extraction and matching of both the needs and availabilities of a variety of resources; various ways to summarize social media content streams; the combination of information from multiple sources; the protection against potential misinformation or other forms of harmful content; the action of adding support for non-English and code-mixed data; and the identification of important images that are posted on social media [12].

It can be found that previous research on social media in natural disasters focuses on the two dimensions of functions and contents, which prove that social media has expanded the channels of risk communication and innovated the ways of risk communication. Risk communication is regarded as an interactive process. Its success ensures individuals’ right to know and influence their attitudes and perceptions of risk. Thus, one of the crucial issues is to understand the individuals’ demand for risk information.

In order to make risk communication more efficient, we must put this demand into a specific context for research. However, relatively little academics do research on the context of natural disaster risk communication on social media. Therefore, this study starts with the exploration of factors influencing successful risk communication by adopting continuance intention to social media usage in natural disasters.

The rainstorm is one of the worst natural disasters in China, which has a wide range of influence, high frequency, and great economic and social losses. Urban rainstorm waterlogging has become a common “urban disease”. Almost every year the plum rain season will appear in different degrees, especially in major cities such as Beijing, Shanghai, Guangzhou, and Wuhan [13]. It is of typical significance to study the risk communication of natural disasters in China by taking the rainstorm disaster as an example.

Among the main social media platforms in China, at present, the Chinese government mainly posts information and interacts with the public based on Weibo (weibo.com, a social media platform similar to Twitter). Up to June 2019, there were 139,270 government microblogs verified by Weibo, including more than 100,000 government agencies below the county level, covering a wide range [14].

Since natural disasters occur year after year, the background of this study requires consideration of the long-term use of information systems (IS) by individuals, and the expectation–confirmation model (ECM) is an important result of previous work on continuance. Other classical theories, such as the theory of reasoned action (TRA) and the theory of planned behavior (TPB), focus on the initial acceptance of IS.

Therefore, this study firstly modified an extended ECM adopted in general IS use context for the suitability of the risk communication in natural disasters. Then, we use SmartPLS (SmartPLS GmbH, Boenningstedt, Germany) to validate the partial least squares structural equation modeling (PLS-SEM) analysis, and the results empirically verify the factors affecting continuance intention to seek information on Weibo during the rainstorm disaster. According to the results, we provide government agencies with practical advice on how to use social media for more efficient risk communication during disasters. At the same time, in the context of applying big data for risk management, this model can also help establish emergency preplans for government agencies to respond to natural disasters.

## 2. Literature Review and Development of Hypotheses

### 2.1. Expectation–Confirmation Model (ECM)

Oliver [15] proposed the expectation confirmation theory (ECT) to study consumer behavior. Based on ECT, the expectation–confirmation model (ECM) is proposed for extensive usage in examining the IS continuance intention. Many empirical studies proved that ECT is predictive in the context of product repurchase as well as service continuance. Bhattacherjee [16] argued that similarity exists between IS users’ continuance decision and consumers’ repurchase decision, and previous IS usage research should be integrated with ECT to form the ECM theory of IS continuance behavior.

Specifically, ECM develops the following theoretical extensions to adapt ECT to the context of IS continuance: (1) The only concern of ECM is post-acceptance variables; (2) ex-post expectation is included in ECT after being amended by ECM; (3) it is cognitively believed that (ex-post) perceived usefulness represents (ex-post) expectation in ECM. The empirical research results of ECM suggested that users’ continuance intention is determined by their satisfaction with IS use and their perception of the usefulness of continued IS use. The expectation confirmed by and usefulness perceived from previous IS use experience will conversely result in user satisfaction.

In addition to the original constructs contained in ECM, Weiyin Hong et al. [17] discussed the role of context value played in the development of IS research theory and expounded on the importance of context-specific models. At first, the constructs of ECM are satisfaction, perceived usefulness, and confirmation. Relevant literatures show that the context and objective of each research determine the choice of independent variables [18,19,20,21].

In the field of continuance intention to use social media, Oghuma et al. [22] employed ECM to the context of mobile instant messaging (MIM) situation. They suggested that the MIM use should include in its unique context perceived service quality, perceived security, perceived enjoyment, and user interface. Research results revealed that in MIM, users’ satisfaction and continuance intention were significantly influenced by perceived service quality as well as perceived usability.

To better understand the characteristics of the social networking service (SNS) user experience, Islam and Mäntymäki [23] added usability to extend IS continuance. Their longitudinal data from 149 LinkedIn users prove that the primary heuristic determining that the user’s subsequent evaluations of usefulness are determined by the prior perceived usefulness, while the confirmation affects the usability. Based on ECM, Chang and Zhu’s [24] research examined perceived social capital and flow experience in users’ intention to use SNSs continually. The results showed that users’ satisfaction as well as their continuance intention are deeply influenced by perceived bridging social capital, rather than bonding social capital. Flow experience will only influence users’ satisfaction, while users’ continuance intention will not be influenced. By using a combination of subjective norm and the expectation–confirmation model, Kim [25] revealed that the user’s continuance intention of SNSs can be predicted by perceived usefulness and perceived enjoyment.

Combining our research context and previous studies, based on the theoretical foundation of ECM, we propose a model of continuance intention to seek information on Weibo during the emergency of the rainstorm. In addition to variables involved in continuance intention to use, a special consideration needs to be given to the initial acceptance of technology by users [26,27,28,29]. In this study, we modify performance expectancy, effort expectancy, social influence, and facilitating conditions in our study.

### 2.2. Development of Hypotheses

#### 2.2.1. Performance Expectancy

Since individuals have a high or “good” self-relevant performance expectancy, Aronson and Carlsmith [30] showed that performance expectancy determines actual performance. In information technology (IT) acceptance research, performance expectancy, which is consistently believed to be the strongest predictor of behavioral intention [26,31], is used to define the degree of benefits consumers gained in using technology to perform certain activities. As for social media, various researches display a positive effect exerted by performance expectancy on behavioral intention [28,32].

Encountering a rainstorm, individuals are usually in a stressful situation, and they will show emotional, cognitive, and behavioral anxieties. In this case, individuals are eager to seek the information of the disaster through various media to reduce the uncertainty. In addition, it is a very convenient channel for them to seek their desired information. When individuals realize that they can get information on Weibo to reduce the impact of a rainstorm on their lives, their performance expectancy will be correspondingly high, so they will act to continuously track disaster information about a disaster on Weibo. Therefore, we proposed the following hypothesis:

**Hypothesis** **1** **(H1).** *Performance expectancy positively influences continuance intention to use.*

#### 2.2.2. Effort Expectancy

The term effort expectancy is used to measure the degree of ease associated with individuals’ use of technology, which relates to perceived ease of use (from Technology Acceptance Model), complexity (from Model of PC Utilization), and ease of use (from Innovation Diffusion Theory) [26]. When a sudden rainstorm happens, individuals are bound to seek information from the most accessible channels. If it is relatively easy to seek relevant information on Weibo, individuals should continue to track disaster information about a disaster on Weibo when effort expectancy is high. Hence, the second hypothesis proposed is that:

**Hypothesis** **2** **(H2).** *Effort expectancy positively influences continuance intention to use.*

#### 2.2.3. Social Influence

According to Venkatesh et al. [26], individuals’ perception of their family members or friends sharing an IT service is termed as social influence. Social influence means that individuals’ tendency to use a certain IT service tends to be influenced by family members, friends, colleagues, and social networks [33]. Social influence can impose an impact on individual behavior through three mechanisms: Compliance, internalization, and identification [34].

Prior studies showed a positive association between social influence and individuals’ behavioral intentions in social media usage [35]. The case of a rainstorm is no exception. If the surrounding individuals tend to seek information on Weibo, that is, when the social influence is high, then the individuals are bound to be affected. As a result, individuals tend to search Weibo for information. Accordingly, we hypothesize:

**Hypothesis** **3** **(H3).** *Social influence positively influences continuance intention to use.*

#### 2.2.4. Facilitating Conditions

Facilitating conditions are defined as the degree of an individual’s belief that the use of the system is supported by an organizational and technical infrastructure [26]. In particular, facilitating conditions determine technology use [31]. In this study, facilitating conditions refer to whether the related social media platform includes proper and enough instructions for users. In other words, if facilitating conditions satisfy users, users may believe that the social media platform enhances their performances [36]. Facilitating conditions directly influence the users’ behavior of using certain information systems.

In the event of a rainstorm, if an individual does not have the conditions to use Weibo, he cannot take relevant actions even if he wishes to seek information on Weibo. However, when conditions are just right, individuals will have the intention to seek information on Weibo. We hypothesize that:

**Hypothesis** **4** **(H4).** *Facilitating conditions positively influences continuance intention to use.*

#### 2.2.5. Satisfaction

Satisfaction is viewed as the most important factor in building and maintaining a long-term consumer loyalty base. Previous studies regard satisfaction as the customer’s overall evaluation of whether a certain service has met their expectation [37]. Satisfaction is also a decisive factor in post-adoption behavior [16,38]. The definition of “satisfaction” emphasizes the fact that an individual’s psychological fulfillment or affective state is closely related to or results from the cognitive appraisal stemmed from the expected performance discrepancy (confirmation). Based on the reasoning drawn from ECM, the operational definition of “satisfaction” is identified as the user’s effect or perception of previous use [16].

With many free social media available in the App Store, satisfaction is very important when a user decides to continue using a particular social media [22,39]. In the context of a rainstorm, the more satisfaction the individual gains when seeking information on Weibo, the higher their continuance intention to resort to Weibo for information will be. Hence:

**Hypothesis** **5** **(H5).** *Satisfaction positively influences continuance intention to use.*

Essentially, both perceived usefulness and performance expectancy measure users’ expected perception of benefits from using information systems. Moreover, perceived usefulness is positively related to satisfaction [16,33,40]. Synthesizing the research model of this paper, it can be expanded. In the case of a rainstorm, individuals may increase their performance expectancy and effort expectancy when they seek information on Weibo. That is, they believe that it is beneficial and easy to seek information on Weibo, so they will naturally have higher satisfaction. Thus, we propose the following two hypotheses:

**Hypothesis** **6** **(H6).** *Performance expectancy positively influences satisfaction.*

**Hypothesis** **7** **(H7).** *Effort expectancy positively influences satisfaction.*

#### 2.2.6. Confirmation

Confirmation is thought to be the extent to which one’s initial expectation is confirmed by their actual use experience [22]. A lot of research revealed that a positive correlation exists between confirmation and satisfaction [19,40,41]. Bhattacherjee [16] explained the process: Lower expectation and/or higher performance results in a greater confirmation, as well as higher customer satisfaction and higher continuance intention, while disconfirmation, dissatisfaction, and discontinuance intention are caused by higher expectation and/or lower performance. Further, disconfirmation was defined as the contradiction between users’ pre-usage expectation of IS usage and actual usage experience [41].

In this study, confirmation will lead to the individual’s satisfaction once their actual experience of using Weibo during the rainstorm matches or exceeds their initial expectation. In contrast, if the actual use of Weibo fails to meet the initial expectation, dissatisfaction will take place. Thus:

**Hypothesis** **8** **(H8).** *Confirmation positively influences satisfaction.*

Satisfaction is viewed as “an additive combination of the expectation level and the resulting disconfirmation” [15]. In Karahanna et al.’s observation [42], as users gain experience with the system, more instrumental considerations (including the efficiency of the innovation to increase one’s job performance) tend to resolve and replace the ease of use concerns. The cognitive dissonance theory believes that confirmation will result in elevated users’ perceived usefulness while disconfirmation reduces perceptions [16]. Existing research proposed that perceived usefulness is significantly affected by confirmation [22,43].

This study specifically investigated individuals who had the experience of seeking information on Weibo during rainstorm disasters. Their previous experience would generate confirmation and modify the previous expectation to the post expectation. Consequently, the higher the confirmation, the higher the performance expectation. Finally, we posit:

**Hypothesis** **9** **(H9).** *Confirmation positively influences performance expectancy.*

**Figure 1:**
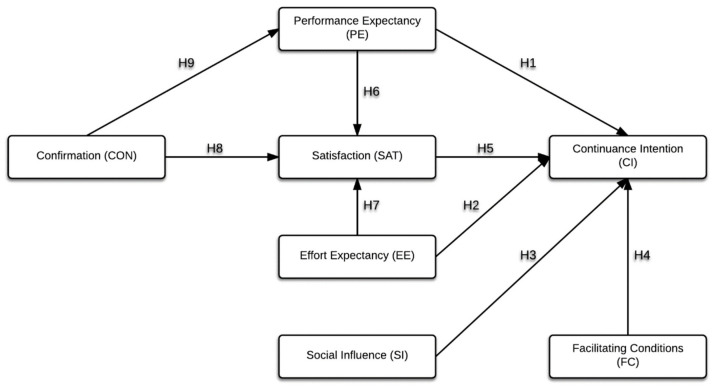
Theoretical model and hypotheses.

The extensive literature review has explored subjects such as PE (abbreviation for Performance Expectancy), EE (abbreviation for Effort Expectancy), SI (abbreviation for Social Influence), FC (abbreviation for Facilitating Conditions), CON (abbreviation for Confirmation), SAT (abbreviation for Satisfaction), and CI (abbreviation for Continuance Intention), as well as the relationship between them. Figure 1 presents the research model and hypotheses.

## 3. Research Methodology

### 3.1. Questionnaire Development

To test the theoretical model, we will first adopt a questionnaire survey with three parts.

The first part was designed to confirm whether the respondents had suffered from the rainstorm and to investigate their Weibo usage habits. In this part, two questions are designed to select samples. High requirements for samples are necessary. Only respondents with the following two experiences are qualified: (1) They have experienced the disaster of a rainstorm, (2) they have sought information on Weibo during the rainstorm disaster. Others whose answers to both questions are “no” will not continue to fill in the questionnaire.

The second part was designed with questions measuring the constructs in the theoretical model. A five-point Likert scale (1 = strongly disagree, 5 = strongly agree) is adopted with multiple items assessing all the constructs, which were inspired by the extant literature and adapted to fit into the research context. The scale items for performance expectancy and effort expectancy were adapted from Venkatesh et al. [26,31] and Talukder et al. [44], while social influence and facilitating conditions were adapted from Min et al. [45] and Zhou et al. [46]. As for continuance intention, satisfaction, and confirmation, they were adapted from Bhattacherjee [16,47], Lee [48], and Oghuma et al. [22] (see Appendix A).

The last is designed with three demographic questions about the participants’ gender, age, and education.

Two experts on risk communication were invited to review the draft. Based on their suggestions, the questionnaire was revised in wording to fit the rainstorm context. And this study was approved by the Academic Committee, School of Journalism and Communication, Wuhan University.

The pilot-test was conducted in Wuhan University by convenient sampling. Of the 50 responses, 28 were qualified who had experienced the disaster of a rainstorm and sought information on Weibo during the disaster. The reliability coefficient was first calculated for the items of each construct, and Cronbach’s α were all greater than 0.7, and some of them were above 0.8. A factor analysis examined that all items had high loadings on their related factors and low cross-loadings on other factors, showing good convergent and discriminate validities.

### 3.2. Data Collection and Sample

To explore the factors affecting individuals’ continuance intention to seek information on Weibo during the rainstorm disasters, the respondents should be “netizens” (who have used the Internet in the past six months [14]), so this study mainly adopts the way of Internet online questionnaire. We inputted the questionnaire into the professional survey website platform—wjx.cn and got the questionnaire link.

The snowball sampling was utilized to recruit respondents. We shared the questionnaire on social media (e.g., WeChat, QQ) through the interpersonal relationship of friends and relatives to find anyone who has sought information on Weibo during the rainstorm disaster. Then, we asked them to invite others with similar experience to join the questionnaire investigation. At the same time, we also used the payment data collection service provided by wjx.cn, to ensure the respondents’ variety and quantity.

We collected a total of 950 responses after excluding incomplete or invalid answers, including 377 valid responses that respondents sought information on Weibo during the rainstorm disaster, and these data were used for the measurement model and structural model evaluation. Table 1 shows the demographic profile of the respondents.

## 4. Results

Partial least squares (PLS) is a powerful second-generation multivariate technique that employs a component-based approach to produce the estimations [49], which has advantages over traditional structural equation modeling (SEM). According to Gefen et al. [50], the minimal sample size based on PLS-SEM should be at least 10 times the number of items in the most complex construct. At the same time, 377 valid responses (more than 250) and more than four indicator variables mean that a large data set has been collected in this study [51]. Meanwhile, the SmartPLS3 software [52] was used to conduct all constructs that are modeled as reflective measures.

### 4.1. Measurement Model

Internal consistency, convergent validity, and discriminant validity [51] are usually used to assess reflective measurement models. Cronbach’s α is usually used to measure the evaluation of the internal consistency reliability. However, Cronbach’s α is more sensitive to the number of items in the construct and considered to be inclined to underestimate the internal consistency reliability [53]. In PLS-SEM, the reliability of each indicator is given priority, so the internal consistency reliability is usually evaluated by composite reliability. As shown in Table 2, the composite reliabilities of latent variables vary from 0.858 to 0.902, all of which are ranging from 0.8 to 0.95. This means it can be regarded as satisfactory. All Cronbach’s α are more than 0.7 threshold values.

As for reflective constructs, the convergent validity is evaluated by the outer loadings as well as the average variance extracted (AVE) [51]. The outer loading of each indicator in this study is above 0.70, and most of the indicators even exceed 0.80, so each reflective indicator in this study can be retained (Table 2). The average variance extracted is defined as the grand mean value of the squared loadings of the indicators associated with the construct. As can be seen from Table 2, the AVE varies from 0.602 to 0.822, all of which are higher than the suggested threshold of 0.5. Thus, all constructs had convergent validity.

Henseler et al. [54] argued that critical examination of the cross-loadings performance as well as the Fornell-Larcker criterion used for the assessment of discriminant validity reveals a lack of reliability of either approach in detecting discriminant validity issues. As a remedy, they proposed to assess the heterotrait-monotrait ratio (HTMT) of the correlations. Argued by Hair et al. [51], the value one for all construct combinations should not be included in the confidence interval of the HTMT statistic. The result of the HTMT presented in Table 3 confirmed the qualified requirement for assessing the discriminant validity.

### 4.2. Structural Model

**Figure 2:**
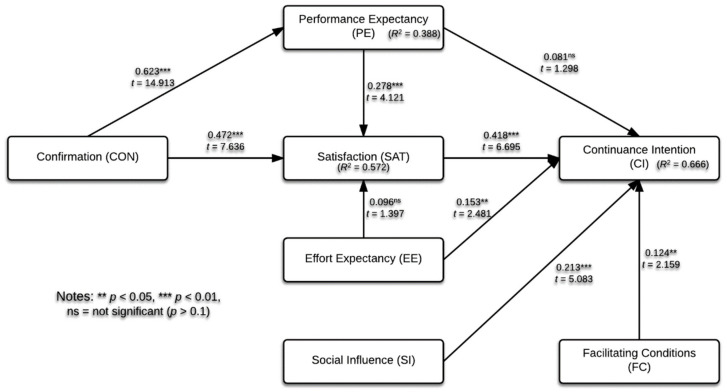
Results of the structural model analysis.

Section 4.1 proves the validity and reliability of the proposed measurement model. In this section, we will proceed to test our proposed hypotheses by using the structural model. Figure 2 is the PLS analysis result. Firstly, the multicollinearity is accessed in terms of the variance inflation factor (VIF), and the results showed that all the estimated values of VIF were smaller than five, ranging from 1.489 to 2.098.

Bootstrapping (5000 resample) is adopted to assess the significance of path coefficients in the structural model. Statistics in Table 4 show the results of the estimation of the structural equations made with PLS. Seven hypotheses (H2, H3, H4, H5, H6, H8, and H7) were supported, two hypotheses (H1 and H7) were rejected. To be more specific, EE, SI, FC, CON, and SAT are proven by the statistics to be significant in the explanation of CI, of which the path coefficient exceeds 0.1. The effect of the SAT on CI is the greatest, as the path coefficient is 0.418. Beyond this, CON has a great positive impact on PE and SAT, the path coefficient of which both are well above the significance level of 0.1. However, PE has a positive impact on CI, but the lower limit of 0.1 is not reached, the impact is not significant, which makes H1 invalid. Similarly, H6 is not supported.

The PLS-SEM method is mainly used for prediction purposes. In our structural model, we use the *R*^2^ values (i.e., coefficients of determination) to represent the amount of explained variance of the endogenous constructs. The *R*^2^ value of our research for CI was 0.666, indicating that the explanatory ability of the proposed model ranges from medium to substantial.

Further, the effect size *f*^2^ value should also be examined. The *f*^2^ value 0.02 is small, 0.15 is medium, and 0.35 is considered strong [55]. The *f*^2^ analysis shows that SAT has a medium effect on CI (*f*^2^ = 0.255), while the *f*^2^ of EE, SI, and FC on CI are small.

The cross-validated redundancy (*Q*^2^) is a means used to assess the structural model predictive relevance and can be calculated by applying the blindfolding method. Scholars concluded that the *Q*^2^ values should be higher than zero [56]. In this study, the *Q*^2^ value for CI is 0.455, which supports the predictive relevance of the model.

As for mediation analysis, the study follows the bootstrapping technique [57], and Table 5 is the result. Statistics in Table 4 show that the relationship from PE to CI is weak (0.081) and nonsignificant (*t* = 1.298; *p* = 0.194). Thus, SAT fully mediates the PE to CI relationship.

Moreover, Hair et al. [51] argued that the GoF can be used to determine whether the model fits in a PLS-SEM context. Being helped by SmartPLS 3, we analyze the value of the standardized mean square residue (SRMR), which should be in a range between <0.08 and 0.1. The SRMR of the structural model is 0.071. It confirms that our model fits well and better explains the factors affecting the individuals’ continuance intention to seek information on Weibo during the rainstorm disaster.

## 5. Discussion

The importance of social media has risen in the field of risk communication. Taking the rainstorm disaster in China as an example, this study analyzes several factors affecting the individuals’ continuance intention to seek information on Weibo. An extended version of the expectation–confirmation model is applied to construct the research model. This model integrates latent variables such as performance expectancy, effort expectancy, social influence, facilitating conditions, confirmation, satisfaction, and continuance intention as determinants of acquisition disaster information using Weibo in the rainstorm. The explanatory power of our proposed research model had an *R*^2^ of 66.6% for continuance intention and an *R*^2^ of 57.2% for satisfaction toward continuance intention.

Our study reveals that among all those factors, satisfaction ranks first in influencing individuals’ continuance intention. The link between satisfaction and intention has been validated in a wide range of other contexts in consumer behavior research [16,22,47,48,58], and its revalidation further proves the robustness of this association. The mediation between performance expectancy and continuance intention is provided by satisfaction.

In the study context, performance expectancy is a kind of cognitive ability, and satisfaction reflects the effect at the individual level. Confirmation can predict both performance expectancy and satisfaction, and the effect is relatively high. When encountering rainstorm, if one’s experience of seeking information on Weibo can meet his expectations, it can enhance the belief in seeking information on Weibo to reduce the impact of disasters on himself. Meanwhile, this experience can also improve his satisfaction.

Since performance expectancy shows a positive and significant effect on satisfaction, we can find that there are two ways to improve satisfaction. In other words, the confirmation of expectations and the tangible benefits of the individual affect the formation of satisfaction in the case of a rainstorm. If both aspects are positive, the continuance intention of seeking information on Weibo can be increased. Satisfaction is based on individuals’ first-hand experience. With a favorable experience of seeking information on Weibo during this rainstorm, the individual will directly and immediately resort to Weibo for information in case of another rainstorm, showing a strong continuance intention. Therefore, the individual’s continuance intention can be simply predicted by evaluating “satisfaction”.

Previous studies argued that social influence is one of the reasons for people to use these technologies in the first place [59]. The results confirm that social influence impacts continuance intention positively and significantly, which suggests that the opinions and recommendations of those important and influential people may motivate individuals to use Weibo for information. Nevertheless, it is a state of emergency to encounter a rainstorm. Social influence may encourage individuals to seek information on Weibo, but this does not mean that they will stay with Weibo after the rainstorm.

Facilitating conditions are also found to play a significant role in the explanation of the continuance intention. When the individual realizes that the existing conditions provide solid technical support and a smooth internet connection, he or she will immediately use Weibo to seek information in the case of a rainstorm, showing positive continuance intention. As reported by the prior studies about social media adoption [28], the findings of effort expectancy also suggest that a lower effort in seeking information on Weibo may result in a higher propensity to adopt Weibo during the rainstorms.

## 6. Conclusions

The study not only extends the research framework of the ECM model but also enriches the research context suitable for the application theory. Factors that will positively and directly affect the individuals’ continuance intention to seek information on Weibo during the rainstorm disaster are effort expectancy, social influence, facilitating conditions, and satisfaction. Among them, satisfaction has the greatest impact. Being directly affected by performance expectancy and confirmation, satisfaction also mediates the balance between performance expectancy and continuance intention.

At the practical level, the findings of this research offer beneficial guidance, enabling government agencies to use social media for more efficient risk communication and establish emergency preplans to respond to the uncertainty during natural disasters.

First, we should attach importance to confirmation in the formation of the individuals’ continuance intention to seek information on Weibo during the rainstorm disaster. Confirmation is the individuals’ perception of the consistency between their expectations and actual experience. In the process of natural disaster risk communication, the government must respond to the individuals’ expectations of the government’s natural disaster risk communication work in social media such as Weibo, which means that the government agency should continuously track the public’s information needs (disaster information, relief supplies, casualties, how to call for help, etc.) in natural disasters by resorting to social media platforms. In the process of drawing up emergency preplans, it is necessary to design different information production and dissemination methods according to different stages and situations of the disaster, focusing on the improvement of confirmation, to timely and accurately disseminate the dynamic information held by the government to the public when the natural disaster outbreaks.

Second, pay full attention to the positive influence of satisfaction on the individuals’ continuance intention to seek information on Weibo. Satisfaction is the individuals’ comprehensive feelings about the use of Weibo to seek information in natural disasters, which is affected by confirmation and performance expectations. In the risk communication of natural disasters, the government agency could respond to public appeals on Weibo promptly and provide practical help to the affected public. This kind of help can start with the basic needs of the affected public, such as posting the missing person column or informing the affected public on how to solve basic living problems such as food, clothing, and transportation. How to respond to these basic information needs of the public needs to be included in the emergency preplans.

Third, it is necessary to consider multiple belief factors which directly affect the individuals’ continuance intention to seek information on Weibo. Furthermore, the government’s emergency preplans for natural disasters must strive to realize intelligent invocation in the event of a sudden disaster. In terms of effort expectancy, the operation of Weibo by government agencies must allow the public to seek information easily and directly, especially in disaster situations of panic. To effectively alleviate the anxiety of the affected public, the voice of government agencies should be timely, accurate, authoritative, and easy to access. In terms of social influence, government departments should not only do a good job in online interaction and information dissemination but also make offline efforts to go deep into the community to make their social media gain the trust of the public. When disaster strikes, these social media can play an effective role in risk communication. Facilitating conditions refers to the construction of information technology infrastructure and the improvement of its resistance against disasters. A smooth mobile Internet connection guarantees the public’s favorable usage experience of social media such as Weibo. In natural disasters, if the mobile Internet infrastructure was easily destroyed, the public cannot get access to any social media, which subsequently results in anything but continuance intention.

Fourth, the government should take the public’s belief into account when compiling the emergency preplans for natural disasters, and develop different solutions to different situations. In practice, the government agencies can adjust their risk communication work according to the public’s belief, achieve intelligent management, and improve the communication efficiency.

This study has some limitations. For example, due to the snowball sampling based on the personal contacts of researchers, the respondents have a higher education level than the whole users of Weibo. One possible assumption is that individuals that do not get a bachelor’s degree may not consciously use Weibo to seek information when facing natural disasters, but this still needs to be explored by future empirical research. In addition to demographic variables, the following research can also classify the population based on the use habits of Weibo, to explore the information-seeking behaviors of different groups in the face of natural disasters. It is important to note that the rainstorm is only one type of natural disaster. Subsequent studies can build on the model of this study and extend the conclusions to different natural disasters.

## Figures and Tables

**Table 1:** Demographic profile of the respondents.

Characteristic		Frequency	Percentage (%)
Gender	Male	153	40.6
	Female	224	59.4
Age	Under 18	0	0
	18–25	185	49.1
	26–30	94	24.9
	31–40	68	18.0
	41–50	25	6.6
	51–60	4	1.1
	Over 60	1	0.3
Education level	Below high school	10	2.7
	College	35	9.3
	Bachelor	179	47.5
	Master	140	37.1
	Doctor	13	3.4

**Table 2:** Factor loadings, Cronbach’s α, composite reliability, and average variance extracted (AVE).

Construct	Items	Outer Loading	Cronbach’s α	Composite Reliability	AVE (%)
Performance Expectancy (PE)	PE1	0.845	0.788	0.876	0.702
	PE2	0.816			
	PE3	0.851			
Effort Expectancy (EE)	EE1	0.791	0.780	0.858	0.602
	EE2	0.765			
	EE3	0.798			
	EE4	0.749			
Social Influence (SI)	SI1	0.886	0.833	0.900	0.822
	SI2	0.826			
	SI3	0.884			
Facilitating Conditions (FC)	FC1	0.844	0.803	0.883	0.715
	FC2	0.839			
	FC3	0.854			
Confirmation (CON)	CON1	0.845	0.805	0.885	0.720
	CON2	0.826			
	CON3	0.874			
Satisfaction (SAT)	SAT1	0.905	0.783	0.902	0.822
	SAT2	0.908			
Continuance Intention (CI)	CI1	0.844	0.816	0.891	0.731
	CI2	0.854			
	CI3	0.868			

**Table 3:** Discriminant validity through the heterotrait-monotrait ratio (HTMT).

Constructs	PE	EE	SI	FC	CON	SAT	CI
PE	0.838						
EE	0.704	0.776					
SI	0.537	0.486	0.866				
FC	0.602	0.648	0.392	0.846			
CON	0.623	0.664	0.611	0.628	0.849		
SAT	0.640	0.606	0.535	0.563	0.710	0.906	
CI	0.646	0.648	0.604	0.592	0.737	0.747	0.855

**Table 4:** Path coefficients (direct effect).

Relationship	Beta	Standard Deviation	*t*-Values	*p*-Values
H1: PE → CI	0.081	0.062	1.298 ^ns^	0.194
H2: EE → CI	0.153	0.062	2.481 **	0.013
H3: SI → CI	0.213	0.042	5.083 ***	0.000
H4: FC → CI	0.124	0.058	2.159 **	0.031
H5: SAT → CI	0.418	0.063	6.695 ***	0.000
H6: PE → SAT	0.278	0.068	4.121 ***	0.000
H7: EE → SAT	0.096	0.069	1.397 ^ns^	0.162
H8: CON → SAT	0.472	0.062	7.636 ***	0.000
H9: CON → PE	0.623	0.042	14.913 ***	0.000

Note: ** *p* < 0.05, *** *p* < 0.01, ^ns^ = not significant (*p* > 0.10) (two-tail).

**Table 5:** Path coefficients (specific indirect effects).

Paths	Beta	Standard Deviation	*t* Values	*p* Values
CON → PE → CI	0.051	0.040	1.284 ^ns^	0.199
EE → SAT → CI	0.042	0.027	1.592 ^ns^	0.112
PE → SAT → CI	0.113	0.030	3.779 ***	0.000

Note: *** *p* < 0.01, ^ns^ = not significant (*p* > 0.10) (two-tail).

## Appendix A. Scale of the Study

**Performance expectancy** [26,31,44]

PE1—When encountering a rainstorm, seeking information on Weibo is convenient and fast.

PE2—When encountering a rainstorm, using Weibo can seek a wealth of information about the disaster.

PE3—When encountering a rainstorm, using Weibo allows me to seek the information I want.

**Effort expectancy** [26,31,44]

EE1—When encountering rainstorm, I can seek information on Weibo skillfully.

EE2—When encountering a rainstorm, Weibo will clearly present the information about the disaster to me.

EE3—Compared with other channels, it is easy to seek information on Weibo in the case of a rainstorm.

EE4—For me, it is easy to learn to seek information on Weibo in the case of a rainstorm.

**Social influence** [45,46]

SI1—People around me would think that I should seek information on Weibo in the case of a rainstorm.

SI2—Many people around me would think that I should seek information on Weibo in the case of a rainstorm.

SI3—People who are important to me would think that I should seek information on Weibo in the case of a rainstorm.

**Facilitating conditions** [45,46]

FC1—I have the knowledge and ability necessary to seek specific information on Weibo at any time.

FC2—I have the resources necessary to seek specific information on Weibo at any time.

FC3—In terms of seeking specific information, Weibo is a very suitable channel for me.

**Confirmation** [16,22,47,48]

CON1—When encountering a rainstorm, my experience with seeking information on Weibo was better than I expected.

CON2—When encountering a rainstorm, my experience with seeking information on Weibo was more convenient than I expected.

CON3—In general, seeking information on Weibo in the case of a rainstorm is in line with my expectations.

**Satisfaction** [16,22,47,48]

SAT1—In general, I am satisfied with my experience seeking information on Weibo in the case of a rainstorm.

SAT2—In general, I successfully sought information on Weibo in the case of a rainstorm.

**Continuance Intention** [16,22,47,48]

CI1—When encountering a rainstorm again, I will also use Weibo to seek information.

CI2—When encountering a rainstorm again, I will first think of seeking information on Weibo.

CI3—I will strongly recommend that others should seek information on Weibo in the case of a rainstorm.
